# Spatial and temporal dynamics of bacterioplankton community composition in a subtropical dammed karst river of southwestern China

**DOI:** 10.1002/mbo3.849

**Published:** 2019-05-06

**Authors:** Shi Yu, Ruoxue He, Ang Song, Yadan Huang, Zhenjiang Jin, Yueming Liang, Qiang Li, Xiaohong Wang, Werner E. G. Müller, Jianhua Cao

**Affiliations:** ^1^ Key Laboratory of Karst Dynamics, MLR & GZAR Institute of Karst Geology Chinese Academy of Geological Sciences Guilin China; ^2^ International Research Center on Karst under the Auspices of UNESCO Guilin China; ^3^ Chengdu Technological University Chengdu China; ^4^ Graduate School of Guilin Medical University Guilin China; ^5^ Environmental Science and Engineering College Guilin University of Technology Guilin China; ^6^ ERC Advanced Investigator Grant Research Group at Institute for Physiological Chemistry University Medical Center of the Johannes Gutenberg University Mainz Germany

**Keywords:** 16S rRNA, bacterioplankton community, dammed karst river, hydro‐physicochemical variability

## Abstract

River damming influences the hydro‐physicochemical variations in karst water; however, such disruption in bacterioplankton communities has seldom been studied. Here, three sampling sites (city‐river section, reservoir area, and outflow area) of the Ca^2+^–Mg^2+^–HCO_3_^−^–SO_4_^2−^ water type in the dammed Liu River were selected to investigate the bacterioplankton community composition as identified by high‐throughput 16S rRNA gene sequencing. In the dammed Liu River, thermal regimes have been altered, which has resulted in considerable spatial‐temporal differences in total dissolved solids (TDSs), oxidation‐reduction potential (Eh), dissolved oxygen (DO), and pH and in a different microenvironment for bacterioplankton. Among the dominant bacterioplankton phyla, Proteobacteria, Actinobacteria, Bacteroidetes, and Cyanobacteria account for 38.99%–87.24%, 3.75%–36.55%, 4.77%–38.90%, and 0%–14.44% of the total reads (mean relative frequency), respectively. Bacterioplankton communities are dominated by *Brevundimonas, Novosphingobium, Zymomonas,* the Actinobacteria hgcIclade, the CL500‐29 marine group, *Sediminibacterium*,* Flavobacterium*,* Pseudarcicella*,* Cloacibacterium,* and *Prochlorococcus*. Their abundances covary with spatial‐temporal variations in hydro‐physicochemical factors, as also demonstrated by beta diversity analyses. In addition, temperature plays a pivotal role in maintaining bacterioplankton biodiversity and hydro‐physicochemical variations. This result also highlights the concept that ecological niches for aquatic bacteria in dammed karst rivers do not accidentally occur but are the result of a suite of environmental forces. In addition, bacterioplankton can alter the aquatic carbon/nitrogen cycle and contribute to karst river metabolism.

## 1 INTRODUCTION

Karst rivers contain the surface networks of water resources for domestic, industrial, and agricultural use and represent an exclusive habitat for microbes that perform critical functions in biogeochemical cycles under the influence of carbonate rock dissolution (Han & Liu, 2004). Karst rivers are commonly regulated by damming, yet the influence of these dams on changes in hydrological series of water discharge is negative or positive (Miao, Ni, Borthwick, & Yang, 2011). Although the diversity and dynamics of microbes in karst springs (Farnleitner et al., 2005; Ohad et al., 2015; Savio et al., 2018), unsaturated and saturated karst aquifers (Cooper et al., 2016; Gray & Engel, 2013; Johnson et al., 2011; Lin et al., 2012; Menning et al., 2018), and water pools (Shabarova et al., 2014) as well as in groundwater‐surface water exchange systems (Li, Song, et al., 2017) have been discussed in the literature, much less attention has been paid to the structure of bacterioplankton communities in dammed karst rivers. In addition, previous studies on bacterioplankton communities in the canyon‐shaped and meso‐eutrophic Rimov Reservoir (Simek et al., 2008), the dammed Ebro River (Ruiz‐González, Proia, Ferrera, Gasol, & Sabater, 2013), and the rivers controlled by the Three Gorges Dam (Huang et al., 2016; Li, Lu, et al., 2017; Yan et al., 2015) did not include the seasonal variation or depth dynamics in bacterioplankton.

Thus, a major challenge in understanding bacterioplankton ecological function is to determine the role of physicochemical properties in dammed karst rivers or the ecological factors that shape bacterioplankton biodiversity and species coexistence (Ávila, Staehr, Barbosa, Chartone‐Souza, & Nascimento, 2017). Despite the controlling factors (such as trophic interactions, evolutionary perspective, spatial heterogeneity, and temporal heterogeneity) of prokaryotic diversity summarized by Torsvik, Øvreås, and Thingstad (2002), the basic principles governing their distribution and abundance in aquatic environments are just beginning to be explored. For instance, Fisher, Klug, Lauster, Newton, and Triplett (2000) highlighted that nutrition (inorganic nitrogen and phosphorus as well as carbon in the form of glucose) and trophic interactions determined bacterioplankton diversity in an oligotrophic lake in northern Wisconsin. Ruiz‐González et al. (2013) used surface water samples from the dammed Ebro River and noted that damming caused a pronounced decline in Betaproteobacteria, Gammaproteobacteria, and Bacteroidetes from upstream to downstream sites, whereas Alphaproteobacteria and Actinobacteria significantly increased after reservoirs were constructed. Ávila et al. (2017) asserted that thermal stratification and oxygen depletion dictated the bacterioplankton diversity in two tropical shallow lakes in the Brazilian Atlantic Forest. Ren et al. (2017) found that spring bacterioplankton community composition shifted significantly under enhanced warming and nutrient‐enriched conditions. Although the above studies provide an exceptional opportunity to gain insight into the controlling factors of bacterioplankton community composition and structure in inland aquatic ecosystems, factors related to bacterioplankton diversity and communities in the city‐river section, reservoir area, and outflow area of dammed karst rivers are still unknown.

The presence of dams is problematic for many aquatic ecosystems (Bednarek, 2001). Consequently, altered thermal regimes in dammed rivers have been observed at a spatial scale (Kelly, Smokorowski, & Power, 2017; Weber et al., 2017). In addition, temperature stratification usually occurs in dammed rivers because of the change to a more reservoir‐like habitat (Bednarek, 2001). It should be noted that temperature can influence the hydrochemistry and recycling of nutrients, *etc*. (Bednarek, 2001; Li, Sun, Han, Liu, & Yu, 2008). Here, we hypothesized that water temperature is the key factor controlling bacterioplankton community composition in dammed karst rivers.

**Figure 1:**
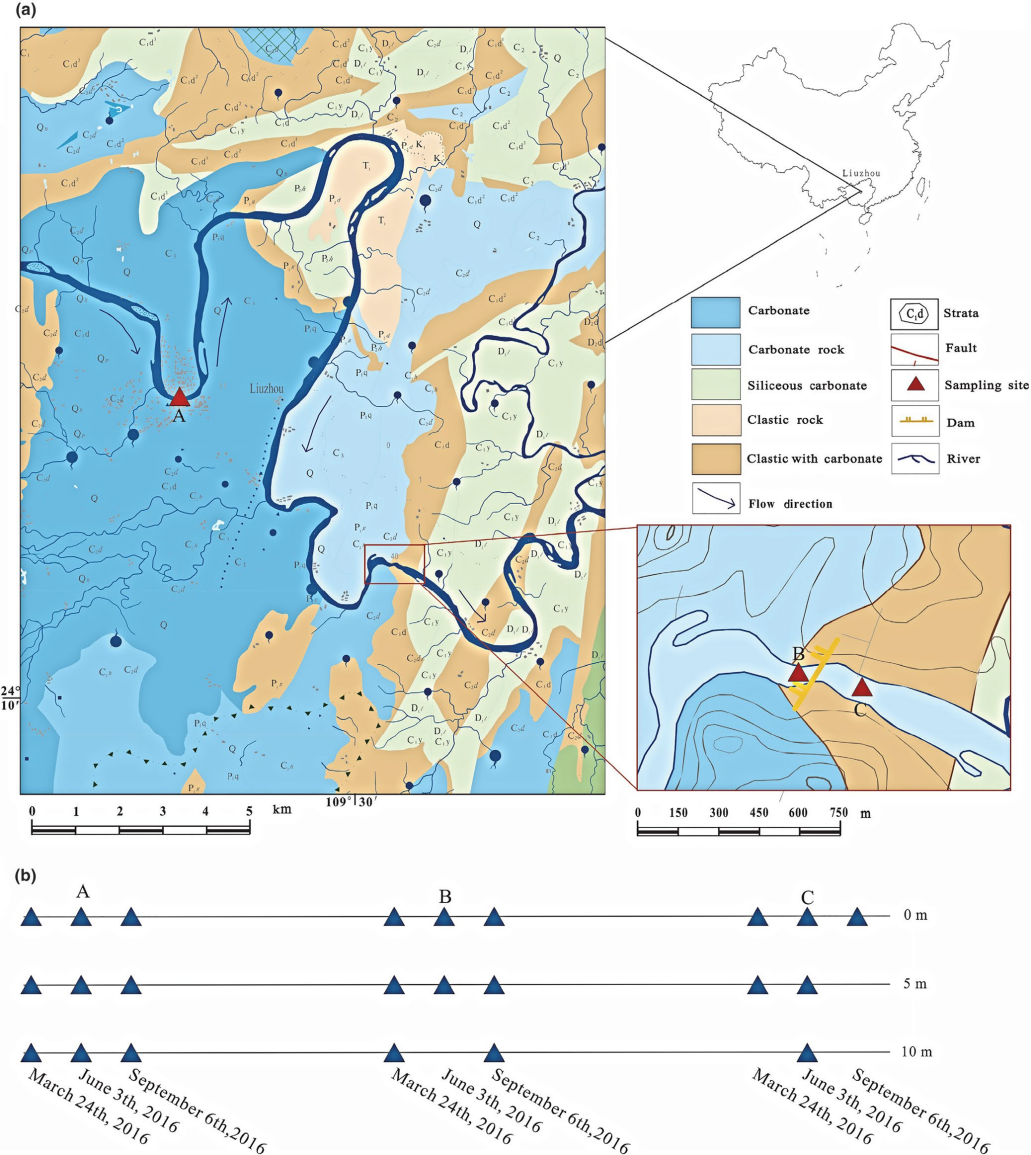
Map showing localization of the dammed Liu River in Liuzhou, Guangxi, P. R. China (a). Timing and depth of sampling locations in the Liu River (b). Sites (A, B, and C) illustrate the sampling locations in the Liu River. The blue triangles indicate the depth of the water samples

As a result, three sampling sites (city‐river section, reservoir area, and outflow area) were selected to investigate bacterioplankton communities, water hydro‐physicochemical properties and their relationship in the dammed Liu River (Figure 1). Consequently, how the bacterioplankton communities changes in relation to hydro‐physicochemical parameters was determined via high‐throughput 16S rRNA gene sequencing.

## 2 METHODS

### 2.1 Study area

The Liu River (24°N‐27°N, 107°E‐111°E) is a tributary within the Pearl River system in Guangxi, China, and was formed by the confluence of the Rong and Long Rivers in Fengshan. The Liu River passes through Liuzhou city (https://en.wikipedia.org/wiki/Liu_River) as well as a sand/shale stone area and limestone area, as indicated in Figure 1. According to water quality monitoring data in China (http://123.127.175.45:8082/), the water environmental quality of the Liu River belongs to class II or III, suggesting that the river can be used as a water resource for domestic use. In addition, under the influence of the East Asian monsoon and South Asian monsoon, 71% of the annual precipitation (1004 mm) occurs between April and August. The mean temperature from December to March is 12.6°C (dry‐cold season), the mean temperature from April to August is 25.5°C (rainy hot season), and the mean temperature from April to August is 22.4°C (dry hot season). In addition, the water flow of the Liu River is controlled by many dams, including a constructed rubber dam in the city of Liuzhou and the Honghua dam (between sampling sites B and C) at the hydroelectric station (Figure 1), resulting in slow water flow and higher nutrient concentrations. From upstream to downstream in dammed Liu River, the sampling site before the rubber dam is named A (city‐river section), the sampling site before the Honghua dam is named B (reservoir area), and the sampling site after the Honghua dam is named C (outflow area).

### 2.2 Sampling procedure and hydrological monitoring

A total of 23 water samples for the analysis of water hydro‐physicochemistry and bacterioplankton community structure were collected in March, June, and September 2016 using a standard water sampler Acc. to Ruttner 2 L (HYDRO‐BIOS, Germany) at three sites in the Liu River (Figure 1). To assess the damming influence on the spatial‐temporal dynamics of the bacterioplankton community composition and hydro‐physicochemistry, water samples were taken at three different depths (0, 5, and 10 m). However, due to water level changes, the samples in the reservoir area and outflow area lacked a layer at 5 and 10 m. Samples were named according to time (M, March; J, June; and S, September), sampling site (A, B, and C), and specific depth (0, 5, and 10 m), in that order (e.g., MA0).

Water samples (approximately 3 L) were prefiltered using 3 μm filter membranes, and then filtered through 0.22 μm pore‐size filter membranes (Merck Millipore, Germany) in situ for bacterioplankton samples. After that, the filter membranes were stored at −80°C until further processing.

**Figure 2:**
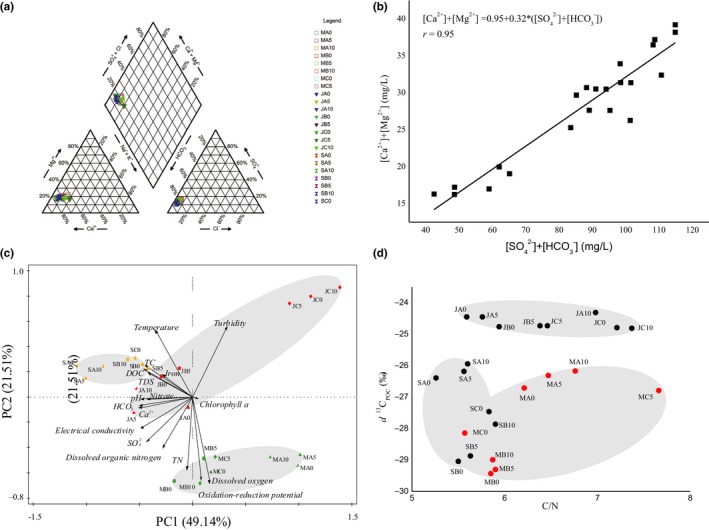
Piper diagram showing the distribution of hydro‐physicochemical data in the dammed Liu River (a). Relationship between [HCO_3_^−^]+[SO_4_^2−^] and [Ca^2+^]+[Mg^2+^] (b). PCA plot displaying hydro‐ physicochemical data (arrows) collected from sampling sites A, B, and C at different depths (0, 5, and 10 m) in March (M), June (J), and September (S) (c). The percentage explained by the axes is shown between parentheses. δ^13^C and C/N ratios of POC in the dammed Liu River (d)

**Figure 3:**
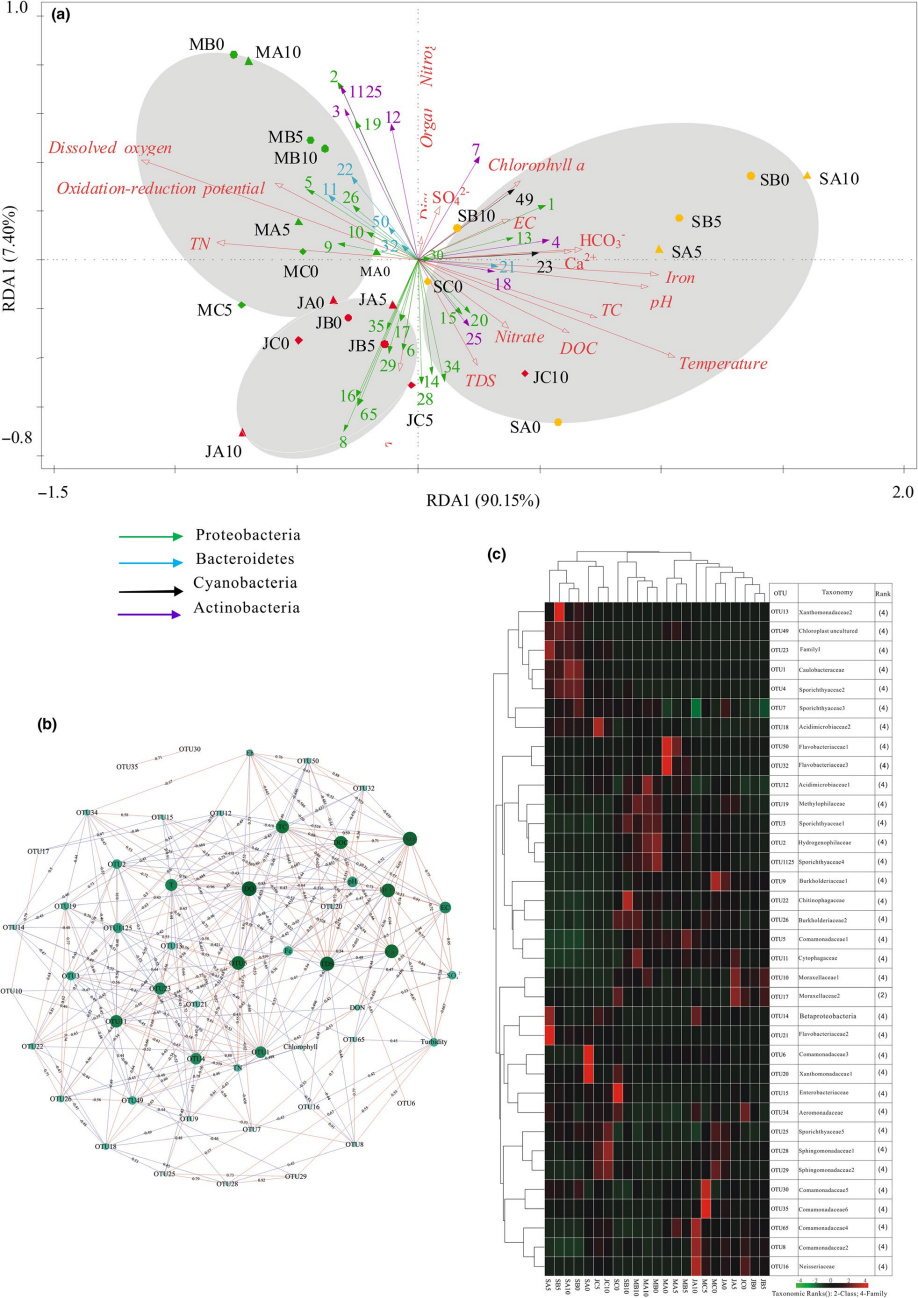
RDA plot used to show the relationship between samples, with the 35 top OTUs (color corresponds to taxonomic affiliation) and environmental variables (red arrows) in the dammed Liu River (a). Correlation network among the OTUs and environmental variables in the dammed Liu River (b). Node size is proportional to the importance. Heat map illustrating the relative frequency of the 35 most abundant OTUs in the dammed Liu River (c)

Water temperature, pH, electrical conductivity (EC), DO, turbidity, chlorophyll‐α (Chlα), dissolved organic nitrogen, TDSs, and Eh were obtained in situ using a multiprobe sensor (YSI, USA). To understand the distributions of ions in Liu River, K^+^, Fe^2+^, Mg^2+^, NO_3_^−^, and SO_4_^2–^ were analyzed by the ICS‐1600 Starter Line IC System (Dionex, USA), and HCO_3_^−^ and Ca^2+^ were titrated in situ using the Aquamerck alkalinity and Hardness test kit (Merck Millipore, Germany), which plots a Piper diagram. The results indicated that the water samples from the Liu River belong to the Ca^2+^–Mg^2+^–HCO_3_^−^–SO_4_^2−^ water type (Figure 2a and b). Samples for total nitrogen (TN), total carbon (TC), dissolved organic carbon (DOC) and dissolved organic nitrogen were collected according to Li, Song, et al. ( 2017) and analyzed using a multi N/C^R^ 3100 total organic carbon (TOC analyzer) (Analytik Jena AG, Germany). Particulate organic carbon (POC) is a broad term that encompasses suspended organic matter such as phytoplankton; consequently, fluvial δ^13^C POC values are a reflection of the relative contributions from freshwater phytoplankton (−25‰ to −30‰) and particulate terrestrial organic matter (−25‰ to −33‰) (Lamb, Wilson, & Leng, 2006). δ^13^C POC was detected in the Institute of Karst Geology, Chinese Academy of Geological Sciences (CAGS), using the Delta V Plus combined with the gas bench automated apparatus (Thermo Company, USA); the error of analysis was better than 0.2 ‰ (1σ). The results were expressed in δ^13^C relative to the Pee Dee Belemnite (PDB) standard, as shown in Figure 3d. The hydro‐physicochemical characteristics of the water samples are summarized in Table 1.

**Table 1:** Water hydro‐physicochemical characteristics in dammed Liu River

	T (°C)	EC (us/cm)	pH	DO (mg/L)	Eh (mV)	Turbidity (NTU)	Chl a (μg/L)	TDS (mg/L)	HCO_3_^−^ (mmol/L)	Ca^2+^ (mg/L)	TC (mg/L)	DOC (mg/L)	TN (mg/L)	DON (mg/L)	Fe^2+^ (mg/L)	NO_3_^−^ (mg/L)	SO_4_^2−^ (mg/L)
MA0	15.41	110.3	6.80	9.98	311.8	29.84	4.40	80	0.95	16.0	1.43	0.84	2.79	2.67	0.01	2.92	7.21
MA5	15.41	105.4	6.80	9.85	311.5	18.50	3.68	80	0.90	17.0	1.46	0.93	2.30	2.29	0.00	2.75	7.15
MA10	15.15	108.8	7.01	10.48	248.4	15.47	4.34	80	0.85	14.0	1.47	0.78	1.47	1.41	0.01	2.69	7.16
MB0	15.32	180.4	7.46	9.75	233.3	4.97	2.12	127	1.25	26.0	2.40	1.77	3.48	3.17	0.01	4.38	12.01
MB5	16.14	178.6	7.04	9.82	263.7	4.74	1.94	127	1.20	25.0	2.55	1.47	2.37	3.37	0.00	4.44	12.01
MB10	16.03	177.8	7.03	9.80	260.6	4.17	2.08	126	1.20	25.0	1.77	1.70	3.58	3.51	0.01	4.69	11.98
MC0	16.14	177.1	6.99	9.12	267.1	4.73	2.34	124	1.30	26.0	1.61	1.53	3.33	3.25	0.00	4.28	11.75
MC5	16.12	176.5	7.03	9.20	270.9	4.71	2.18	123	1.35	26.0	1.58	1.31	2.58	2.79	0.00	4.59	11.77
JA0	23.84	163.8	7.58	8.34	264.4	23.88	1.43	172	1.50	22.5	2.38	2.36	2.93	2.78	0.01	5.05	9.86
JA5	24.26	162.7	7.68	8.09	231	28.33	1.44	171	1.30	24.0	3.82	3.47	3.28	4.06	0.01	5.09	9.77
JA10	24.27	163.7	7.65	8.25	223.3	28.91	1.57	171	1.40	24.0	3.77	3.58	2.62	2.69	0.00	5.16	9.89
JB0	24.61	177.1	7.47	7.81	206.8	94.01	2.02	181	1.40	30.0	2.51	1.90	2.31	2.30	0.00	5.19	12.94
JB5	24.58	154.9	7.52	7.91	211.8	113.19	2.51	167	1.20	22.0	3.31	2.06	2.50	2.21	0.00	4.83	10.30
JC0	26.42	89.91	7.34	7.75	210.2	290.88	2.51	129	0.70	15.0	2.36	1.42	2.05	2.01	0.01	3.48	5.95
JC5	26.73	88.73	7.13	7.45	222.6	249.31	2.46	127	0.70	14.0	2.61	2.06	2.11	1.85	0.02	3.70	5.91
JC10	24.08	86.90	7.14	7.70	222.8	322.77	2.68	127	0.60	14.0	1.26	1.07	1.24	2.12	0.01	3.83	5.88
SA0	27.79	191.80	7.76	6.16	199.8	9.51	1.43	144	1.70	34.5	3.64	2.87	2.15	2.78	0.09	5.39	11.17
SA5	27.62	191.80	7.83	6.65	216.9	9.23	1.64	144	1.70	33.5	3.60	3.04	1.72	4.06	0.09	5.30	11.16
SA10	27.62	192.50	7.79	6.59	215.6	11.63	1.59	144	1.60	32.5	2.97	2.84	1.91	2.69	0.10	5.32	11.15
SB0	30.88	167.4	8.02	6.66	233.9	3.62	6.09	134	1.50	27.0	3.51	2.57	1.51	2.30	0.01	4.03	10.04
SB5	29.1	166.1	7.96	6.67	231.6	3.93	5.48	135	1.45	27.0	3.62	2.10	1.51	2.21	0.01	4.00	10.05
SB10	29.86	165.8	8.02	6.50	221.5	4.15	4.60	135	1.65	28.0	3.19	2.88	1.38	2.01	0.01	4.03	10.05
SC0	29.14	186.8	7.92	6.53	222.9	3.85	3.05	137	1.60	32.0	3.08	2.80	1.39	1.85	0.01	4.20	10.62

### 2.3 DNA extraction and HiSeq

DNA extraction was performed using the PowerWater^®^ DNA Isolation Kit (Mobio Laboratories, Inc., Carlsbad, CA, USA) following the manufacturer's instructions.

For HiSeq sequencing, PCR primers 515F (5′‐CTACCGATTGCGGTGYCAGCMGCCGCGGTA‐3′) and 909R (5′‐CCCCGYCAATTCMTTTRAGT‐3′) were used to amplify the V4‐V5 region of 16S rRNA genes (Tamaki et al., 2011). The PCR products targeting the V4‐V5 region of 16S rRNA genes were purified using the TIANquick Maxi Purification Kit [TIANGEN Biotech (Beijing) Co., Ltd, China]. Then, 16S rRNA gene sequencing was performed on the Illumina HiSeq 2500 platform (Illumina Inc., San Diego, CA) at the Chengdu Institute of Biology, Chinese Academy of Sciences.

### 2.4 Bioinformatics analysis and statistical analyses

The achieved 16S sequence data from 23 water samples were processed using the QIIME 1.7.0 software (Kuczynski et al., 2012; Li et al., 2018). Low‐quality sequences with lengths below 150 bp and an average quality score below 30 were excluded. In addition, sequences matching plant chloroplast or mitochondrial 16S rRNA were also filtered and rarefaction of all samples was done on the reader of 3342. Representative sequences from each operational taxonomic unit (OTU) cluster at the 97% similarity level were aligned with the PyNAST aligner to the SILVA128 ribosomal RNA databases. Consequently, 590551 low‐quality reads generated 76866 high‐quality reads grouped into 5979 OTUs. The coverage ranged from 95.06% to 98.47%. Bootstrap OTU richness, Chao 1 estimates, the inverse Simpson index (i.e., a measure of evenness) and Simpson diversity index were calculated on rarefied OTU tables to assess the distribution patterns of bacterioplankton communities’ OTUs. Beta‐diversity measures (unweighted UniFrac and Bray–Curtis distances) were visualized using principal coordinate analysis plots generated with the EMPeror software package to assess the similarity in OTU structure among different bacterioplankton communities (Vázquez‐Baeza, Pirrung, Gonzalez, & Knight, 2013). The Partial Mantel test based on Pearson's product‐moment correlation was applied to explore the correlations among bacterioplankton communities (main phyla with the relative abundance > 1%), temperature, pH, DO, and nutrition factors (HCO_3_^−^, Ca^2+^, DOC, TN, DON, Fe^2+^, NO_3_^−^, SO_4_^2−^) using PASSaGE 2 so as to eliminate collinearity between variables (Yao et al., 2017). Moreover, the function inner plot in R package Partial least squares‐path modeling (PLS‐PM) (Sanchez, 2013) was applied to construct the model for exploring the relationships among bacterioplankton communities, alpha diversity, temperature, pH, DO, and nutrition factors, which can help us to visually inspect the model defined for the path matrix. Correlation networks were used to detect the interactions among the 35 most abundant OTUs and between these OTUs and hydro‐physicochemical variables (Ávila et al., 2017). Their interactions with *p* values <0.05 based on Pearson's product‐moment correlation were visualized and customized using Gephi 0.9.2 (Barberán, Bates, Casamayor, & Fierer, 2012). The detailed data of 16S rRNA gene sequencing are summarized in Table S1.

Moreover, correlation analyses were performed using the Pearson correlation method to detect the relationship between hydro‐physicochemical factors and bacterioplankton as well as Tukey test were proposed to reveal significant differences with SPSS 13.0 software for Windows XP (IBM, Armonk, NY, USA). Principal component analysis (PCA) was used to investigate hydro‐physicochemical characteristics and redundancy analysis (RDA) was used to detect the strength of hydro‐physicochemical factors upon bacterioplankton communities’ structure and OTU associations with samples.

## 3 RESULTS AND DISCUSSION

### 3.1 Hydro‐physicochemical characteristics of dammed Liu River

**Figure A1:**
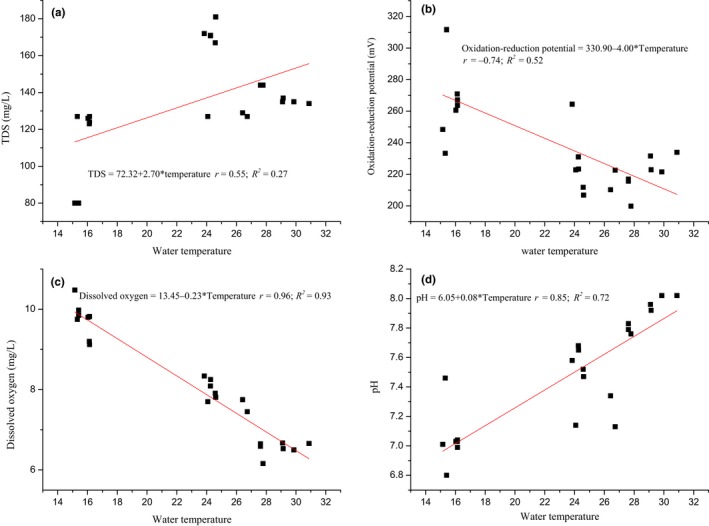
Change in TDS, oxidation‐reduction potential, dissolved oxygen, and pH with water temperature

The spatial‐temporal hydro‐physicochemical characteristics of the dammed Liu River are listed in Table 1 and Figure 2. [Ca^2+^]+[Mg^2+^] are the major cations with a molarity percentage of 79.24%~95.09%; [HCO_3_^−^]+[SO_4_^2−^] are the major anions with a molarity percentage of 85.30~94.33%, which reflected the combined effect of limestone weathering (Li et al., 2008) and acid precipitation ([Ca^2+^ + Mg^2+^]/[HCO_3_^−^ + SO_4_^2−^]≈0.95) (Gao et al., 2009). Although water column thermal stratification is not evident, the spatial‐temporal dynamics of water temperatures are clear. In dammed rivers, water temperature usually increases from upstream to downstream, resulting in changed thermal capacities (Hanna, Saito, Bartholow, & Sandelin, 1999). In addition, for strongly seasonal rivers with varying water temperatures, 26% of the variation in water temperature is attributed indirectly to low flow changes, and the remaining fraction is attributed directly to changed atmospheric energy input (van Vliet et al., 2013). Consequently, the temperature can be clustered into three groups as seen in Figure 2c. In addition, water temperature can affect the Ca^2+^–Mg^2+^–HCO_3_^−^–SO_4_^2−^ system (Beck, Grossman, & Morse, 2005; Dreybrodt, 2012). pH, DO, TDS, and Eh covary with temperature behavior (Pearson's *r *=* *0.854, −0.964, 0.552, and −0.739, respectively, *p *<* *0.001, *n* = 23, two‐tailed) (Figure A1). Water temperature also can affect bacterioplankton growth, resulting in oxygen uptake and bacterioplankton production (determining the accumulation of POC and newly produced DOC) (Lindström, Kamst‐Van Agterveld, & Zwart, 2005; Søndergaard, Borch, & Riemann, 2000). In this respect, DO has a significantly negative relationship with TC and DOC in the dammed Liu River (Pearson's *r *=* *−0.715 and −0.687, respectively, *p *<* *0.001, *n* = 23, two‐tailed). Thus, δ^13^C and C/N ratios of POC indicate that bacterioplankton production is relating to water temperature differences (Figure 2d), which confirms the findings that water temperature and oxygen have strong positive correlations with bacterioplankton (Araújo & Godinho, 2008).

To better investigate the influence of hydro‐physicochemical factors on bacterioplankton community structure, RDA plots revealed that bacterioplankton's OTU distribution across all samples could be mainly explained by the RDA1 axis (*p *=* *0.028), significantly correlating with water temperature (*p *=* *0.002), pH (*p *=* *0.002), DO (*p *=* *0.002), and iron (*p *=* *0.004) (Figure 3a). Notably, iron can directly limit bacterial growth; thus, in turn, bacterioplankton account for up to 70% of the total iron uptake in aquatic environments (Arrieta, Weinbauer, Lute, & Herndl, 2004; Gledhill et al., 2004). Moreover, hydro‐physicochemical variation strongly correlated with Proteobacteria‐, Bacteroidetes‐, Cyanobacteria‐, and Actinobacteria‐related OTU clusters, suggesting that environmental variables had an important influence on the clustering of taxonomically related OTUs (Figure 3a and c). The network analysis showed associations between co‐occurring OTUs and environmental variables (Figure 3b), which were proven by the links between environmental drivers and tribe responses (Newton, Jones, Eiler, McMahon, & Bertilsson, 2011). Thus, the results support the notion that bacterioplankton in the dammed Liu River are not there accidentally but are the result of a suite of environmental forces.

### 3.2 Spatial‐temporal variations in bacterioplankton community composition

**Figure 4:**
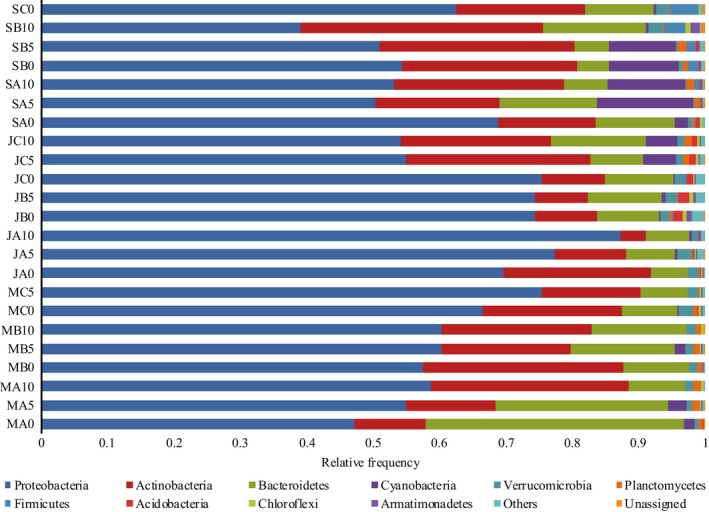
Comparison of the quantitative contribution of the sequences affiliated with different bacterial phyla to the total number of sequences from the water samples. Sequences not classified to any known phylum are included as unassigned bacteria. In each water sample, bacterial phyla with a largest relative frequency of less than 1% are included as others

Of the reads, 96.35% were assigned to 10 major phyla, including Proteobacteria, Actinobacteria, Bacteroidetes, Cyanobacteria, Verrucomicrobia, Planctomycetes, Firmicutes, Acidobacteria, Chloroflexi, and Armatimonadetes, as illustrated in Figure 4 (Archaea are not included). Among these bacterioplankton phyla, Proteobacteria, Actinobacteria, Bacteroidetes, and Cyanobacteria account for 38.99%–87.24%, 3.75%–36.55%, 4.77%–38.90%, and 0%–14.44% of the total reads (mean relative frequency), respectively. These phyla have also been found in different relative proportions in other freshwater ecosystems worldwide (Ávila et al., 2017; Newton et al., 2011). Notably, if a “core” assemblage as the subset of OTUs is present in all samples, then this assemblage can be defined as a core OTU (Engel, 2010). Interestingly, in our study, only 2.31% of all OTUs (24 core OTUs) are shared among all 23 samples. The largest group of core OTUs are Betaproteobacteria (10 OTUs), followed by Actinobacteria (6 OTUs) and Alphaproteobacteria (4 OTUs). Most of these OTUs were related to strains or sequences obtained from various aquatic environments, especially karst groundwater (Engel, 2010).

**Figure A2:**
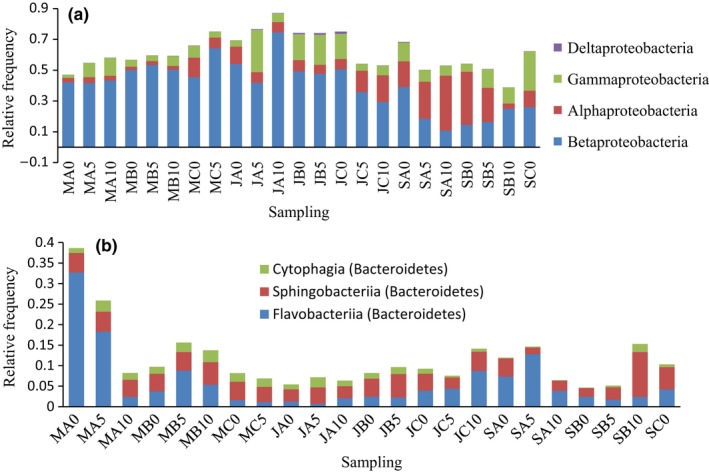
Comparison of the quantitative contribution of the sequences affiliated with different class

Proteobacteria are dominant in all water samples and mainly consist of the classes Alphaproteobacteria, Betaproteobacteria, Deltaproteobacteria, and Gammaproteobacteria. Betaproteobacteria (accounting for 10.77%–74.58%) constitute the largest fraction of the bacterioplankton community in the Liu River, as shown in Figure A2. They have also been found to be the numerically dominant group in other freshwater ecosystems (Newton et al., 2011). This result is in accordance with the classification of Betaproteobacteria as r‐strategists (Novello et al., 2017), that is, taxa able to grow rapidly under conditions of high resource availability. In addition, the abundance of Proteobacteria increases with depth in March and June (except for JC0, JC5, and JC10); however, the abundance of Proteobacteria decreases with depth in September. Betaproteobacteria have a similar variation pattern, though they are often the most abundant bacteria inhabiting the upper waters of lakes (Newton et al., 2011). Previous studies demonstrated that Proteobacteria are involved in a variety of biogeochemical processes in aquatic ecosystems (Damashek & Francis, 2018; Xu et al., 2014; Zhang et al., 2015). Thus, they are more active due to a greater availability of POC and DOC at that depth, as seen in Figures 2d and 3a, which demonstrates that depth has a significant effect on their abundance in freshwater (Gattuso, Peduzzi, Pizay, & Tonolla, 2002). Notably, a significant increase in Proteobacteria occurs during the rainy, hot season (June), which is generally a period of increased grazing by phagotrophic protists (Newton et al., 2011). Among the most frequent Proteobacterial OTUs, OTUs 1, 28, and 29 are classified into Alphaproteobacteria; OTUs 2, 5, 8, 6, 9, 14, 16, 19, 26, 30, 35, and 65 are classified into Betaproteobacteria; and OTUs 10, 13, 15, 17, 20, and 34 are classified into Gammaproteobacteria. Alphaproteobacteria are at the hub of the global nitrogen cycle (Newton et al., 2011). Indeed, the genus *Brevundimonas* (OTU 1) has a small angle with dissolved organic nitrogen and nitrate, as indicated in Figure 3a, suggesting that they are nitrogen‐fixing bacteria (Liu, Peng, & Li, 2012). In addition, the genera *Novosphingobium* (OTU 28) and *Zymomonas* (OTU 29) have small angles with turbidity. In previous studies, *Novosphingobium* was often isolated from humic‐rich subsurface water (Glaeser et al., 2013; Hutalle‐Schmelzer, Zwirnmann, Krüger, & Grossart,^2010^), and sugar is an important factor for *Zymomonas* cells (Sulfahri, Amin, Sumitro, & Saptasari, 2016), which can be reflected by water turbidity (Lind et al., 1992). In contrast to Alphaproteobacteria‐related OTUs, Betaproteobacteria‐related OTUs 5, 6, 65, and 30 belong to the family Comamonadaceae; Betaproteobacteria‐related OTUs 2 and 16 belong to the families Hydrogenophilaceae and Neisseriaceae; Betaproteobacteria‐related OTUs 8 and 35 belong to the genus *Variovorax*; Betaproteobacteria‐related OTUs 9 and 26 belong to the genus *Polynucleobacter*; Betaproteobacteria‐related OTU 19 is affiliated with clade OM43; and Betaproteobacteria‐related OTU 14 is affiliated with an unknown order. RDA revealed that depth, pH, carbon substrate preferences, and time factors are closely related to Betaproteobacteria‐related OTUs, as confirmed by Figure 3a. Although the Gammaproteobacteria, like Alphaproteobacteria, are not particularly abundant in freshwater lakes, Gammaproteobacteria‐related OTUs are positively correlated with nutrient availability, as indicated by the small angles among OTU 10 and TN, among Gammaproteobacteria‐related OTUs 17 and 34 and water turbidity, among Gammaproteobacteria‐related OTUs 15 and 20 and nitrate as well as among Gammaproteobacteria‐related OTU 13 and dissolved organic nitrogen, which prove that members of the Gammaproteobacteria exhibited even faster growth rates when nutrition was added to enclosures (Gasol et al., 2002; Newton et al., 2011). Moreover, the heat map showed that co‐occurring Proteobacteria‐related OTUs can be classified into three groups according to the spatial‐temporal variations, as confirmed by Figure 3a.

The phylum Actinobacteria is often the numerically dominant phylum in lakes, where it can account for 50% of the bacteria in the surface waters and is present in the bottom waters of lakes (Newton et al., 2011). However, Actinobacteria account for only a moderate fraction in the Liu River, a result that is similar to the observed results in Carioca and Gambazinho lakes (Ávila et al., 2017). The abundance of Actinobacteria has no obvious pattern with depth; however, we noted a significant increase during the dry, cold season (March), which, in general, is a period without high incident solar UV radiation and extreme daily temperature changes (Dorador, Vila, Witzel, & Imhoff, 2013). Similarly, in Lakes Chungará and Cotacotani, the relative abundance of Actinobacteria was also high in the dry, cold season than that in the wet season (Aguilar, Dorador, Vila, & Sommaruga, 2018). Moreover, the abundance of Actinobacteria often decreases with decreasing oxygen concentrations (Newton et al., 2011) and pH has been identified as another driver of the Actinobacteria clade and tribe distribution (Newton et al., 2011). In contrast, in this study Actinobacteria have no significant correlations with DO and pH. Besides that, our study showed that among Actinobacteria, OTUs 3, 4, 7, 25, and 1125 affiliated with the hgcIclade as well as OTUs 12 and 18 affiliated with the CL500‐29 marine group are always the most abundant OTUs in the Liu River. The presence of the CL500‐29 marine group was surprising, as it has been found primarily in marine ecosystems, while through the work of Zwart and others (Zwart, Crump, Kamst‐van Agterveld, Hagen, & Han, 2002), the CL500‐29 marine group was determined to exist in freshwater rivers and lakes. Moreover, OTUs 4 and 18 are positively correlated with pH; OTUs 3, 7, 12, and 1125 are positively correlated with dissolved organic nitrogen; and OTU 25 is positively correlated with nitrate, as indicated by their small angles in Figure 3a, suggesting that the abundance and distribution of the freshwater Actinobacteria are related to the chemical–physical properties of the Liu River. In addition, members of the hgcI clade belonging to the phylum Actinobacteria are known to have a competitive advantage over others in lakes that are characterized by low DOC and low temperature (Glöckner et al., 2000).

Bacteroidetes have colonized many different ecological niches, including freshwater, where they display various biological functions. Although Ávila et al. (2017) detected few Bacteroidetes in Carioca and Gambazinho lakes, in this study, there are three distinct classes within the Bacteroidetes: Flavobacteriales, Cytophagales, and Sphingobacteriales (Figure A2). Interestingly, Bacteroidetes, which have the propensity to occur during periods or at sites characterized by high external DOC loading (Newton et al., 2011), have a significantly negative correlation with DOC (Pearson's *r *=* *−0.445, *p *=* *0.034, *n* = 23, two‐tailed). O'Sullivan, Rinna, Humphreys, Weightman, and Fry (2006) noted that Bacteroidetes are involved in organic carbon cycling, particularly in terms of the utilization of high molecular mass dissolved organic matter in nutrient‐rich aquatic habitats. Consequently, fast‐growing Bacteroidetes are related to elevated concentrations of DOC (Ruiz‐González et al., 2013). In addition, Bacteroidetes‐related OTU 22 is classified as *Sediminibacterium* (Sphingobacteriales), OTUs 50 and 32 are classified as *Flavobacterium* (Flavobacteriales), OTU 11 is classified as *Pseudarcicella* (Cytophagaceae), and OTU 21 is classified as *Cloacibacterium* (Flavobacteriales). *Cloacibacterium* (OTU 21) was positively correlated with TC and DOC, and *Sediminibacterium* (OTU 22), *Flavobacterium* (OTUs 50 and 32) and *Pseudarcicella* (OTU 11) were negatively correlated with TC and DOC, as indicated by their small angles in Figure 3a, suggesting that Flavobacteriales, Cytophagales, and Sphingobacteriales have different organic carbon utilization rates and sizes (Reintjes, Arnosti, Fuchs, & Amann, 2017) that have previously been overlooked. In addition, *Cloacibacterium* (OTU 21) is positively correlated with iron (Figure 3a), suggesting that *Cloacibacterium* growth is limited by iron (Arrieta et al., 2004; Gledhill et al., 2004).

Cyanobacteria are the largest and most widely distributed group of photosynthetic prokaryotes, found in ecosystems ranging from marine and freshwater to terrene (Stanier & Bazine, 1977). Interestingly, Cyanobacteria account for only a small fraction and are hardly detected in MA10 and MB10. In addition, the Cyanobacteria abundance increases with depth (except for MC and SB), and their abundances are higher in March and September than in June. This observation was supported by the fact that river damming leads to the disappearance of cyanobacterial blooms (Domingues, Barbosa, & Galvão, 2014) and that cyanobacterial growth is usually enhanced by high water residence times during the dry, cold season and dry, hot season with low freshwater flows (Domingues, Barbosa, & Galvao, 2005). Under these conditions, Cyanobacteria can grow abundantly and form extensive blooms, as confirmed by Figure 2d. The freshwater Bacteroidetes are often found during periods following cyanobacterial blooms; however, no correlation between the freshwater Cyanobacteria and Bacteroidetes (Pearson's *r *=* *−0.112, *p *=* *0.610, *n* = 23, two‐tailed) is observed in our study, casting doubt on their relationship that has been reported (Newton et al., 2011). However, freshwater Cyanobacteria have a significantly negative correlation with Proteobacteria (Pearson's *r *=* *‐0.486, *p *=* *0.048, *n* = 23, two‐tailed). The relationships between Cyanobacteria and Alphaproteobacteria, Betaproteobacteria, Deltaproteobacteria as well as Gammaproteobacteria are also discussed. Notably, Cyanobacteria have a significantly negative correlation with Alphaproteobacteria (Pearson's *r *=* *−0.769, *p *=* *0.00, *n* = 23, two‐tailed) and a significantly positive correlation with Betaproteobacteria (Pearson's *r *=* *0.864, *p *=* *0.00, *n* = 23, two‐tailed). Some Cyanobacteria are able to produce potent toxins and have drastic impacts on the ecosystem and surrounding communities (Steffen et al., 2012). Consequently, cyanobacterial blooms will disrupt aquatic food webs and act as a driver of hypoxia, especially changing the sensitivity of Proteobacteria to grazing pressure (Eiler, Olsson, & Bertilsson, 2006). As exposed, common freshwater lake genera belonging to Cyanobacteria include *Microcystis*,* Anabaena*,* Aphanizomenon*,* Oscillatoria*,* Planktothrix*,* Synechococcus*, and *Cyanothece* (Newton et al., 2011); however, in our study, the top Cyanobacteria‐related OTUs 23 and 49 are classified into *Prochlorococcus*. *Prochlorococcus* (OTUs 23 and 49) are positively correlated with Chlα, as indicated by their small angles in Figure 3a, as previously reported by Domingues et al. (2014). The results suggest that they might contribute significantly to global primary productivity through oxygenic photosynthesis (Boekema et al., 2001; Newton et al., 2011; Stanier & Bazine, 1977). In addition, *Prochlorococcus* has small angles with dissolved organic nitrogen and nitrate, suggesting that they can play a key role in nutrient cycling in freshwater (Stanier & Bazine, 1977). Cyanobacteria‐related OTU 23 is positively correlated with iron (Figure 3a), which is supported by the iron limitation of *Prochlorococcus* sp. (Mann & Chisholm, 2000).

### 3.3 Spatial‐temporal variations of bacterioplankton community diversity

To investigate the effects of spatial (sampling site and depth) and temporal (season) changes on bacterioplankton communities, we examined alpha and beta diversity (Figure 4 and Appendix Table A1). According to the numbers of observed and estimated OTUs as well as Shannon and Simpson diversity in the Liu River, alpha diversity shows highly spatial‐temporal variations, however, alpha diversity measures have no significant difference with depths. The Shannon and Simpson diversity measures in outflow area have the significant difference with these measures in city‐river section and reservoir area. As to the season changes of bacterioplankton communities, the numbers of estimated OTUs (Chao 1) as well as Shannon and Simpson diversity in June have the significant difference with them in March and September. In this respect, rapid decreases in alpha diversity usually appear in March and September. Although a two‐tailed Pearson correlation showed that TDS has a significantly positive correlation with Chao1 and the observed OTUs (*r *=* *0.557 and 0.597, respectively, *p *<* *0.01, *n* = 23), Eh has a significantly negative correlation with the observed OTUs (*r *=* *−0.462, *p *<* *0.05, *n* = 23), and turbidity has a significantly positive correlation with the Shannon and Simpson indexes and observed OTUs (*r *=* *0.591, 0.468, and 0.497, respectively, *p *<* *0.05, *n* = 23), a one‐tailed Pearson correlation indicated that temperature also has a significantly positive correlation with the Shannon index and observed OTUs (*r *=* *0.353 and 0.354, respectively, *p *<* *0.05, *n* = 23), and DO has a significantly negative correlation with the Shannon index (*r *=* *−0.368, *p *<* *0.05, *n* = 23). Indeed, Ávila et al. (2017) found that DO showed a significantly negative correlation with the Shannon index in two tropical shallow lakes in the Brazilian Atlantic Forest, as indicated by regression analysis; however, the regression results about DO and the Shannon index are not significant in our study (*R* ^2^ = 0.368, *p *=* *0.084).

Moreover, higher alpha diversity is usually found at the surface water in the JB and JC samples, whereas the JA sample has higher alpha diversity at a depth of 5 m. Although alpha diversity measures have no significant difference with depths (Appendix Table A1), the high alpha diversity values of the JA5 sample may be attributed to a less stressful environment due to higher nutrient availability and isolation from external disturbances, such as UV radiation, wind, and waves at this layer (Ávila et al., 2017). In contrast, the alpha diversity values are lower in the surface and bottom layers of the MB0 and SA10 samples, suggesting that anthropogenic activity (e.g., shipping activity, fishing or swimming) can decrease bacterioplankton diversity in the surface layer, and the input of sand/mud restricts supplemental energy generation by light harvesting for bacterioplankton (Gómez‐Consarnau et al., 2007), as confirmed in Table 1. Compared with site C, site A and B under the impact of a long water‐retention time is quite stable with slow rates of water flow, high water transparency and high nutrient levels, which in turn enhance the difference of bacterioplankton (Yang et al., 2018), as seen in Appendix Table A1. Interestingly, in our study, the alpha diversity with minimal spatial‐temporal variations in other C samples may be attributed to the influence of water discharge of the Honghua dam resulting in a normalized bacterioplankton community; however, the hydro‐physicochemical characteristics of site C are different. Moreover, because site C and B are directly connected along the Honghua dam, the JC0 sample also has high alpha diversity values.

**Figure 5:**
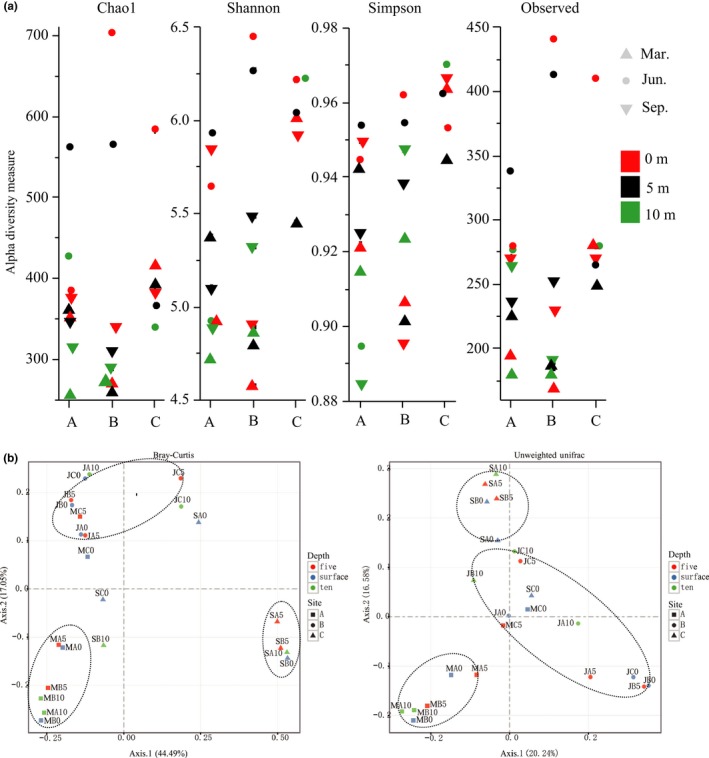
Alpha diversity of the bacterioplankton communities of the Liu River (a). PCA plots of bacterioplankton community structure based on the unweighted UniFrac and Bray–Curtis distances (b)

On the basis of the fact that bacterioplankton community members turn over quickly in response to changing environmental conditions and beta diversity is the variation in species composition among sites in a geographic area (Legendre, Borcard, & Peres‐Neto, 2005), we used the unweighted UniFrac and Bray–Curtis distances of beta diversity, independent of changes in alpha diversity, to compare the range of bacterioplankton diversity in spatial‐temporal variations (Figure 5). Highly similar communities (three clusters) are observed at the same sampling time, suggesting that a mixed seasonal environment can facilitate bacterial coexistence (Huang, Dong, Jiang, Wang, & Yang, 2016), as confirmed by Appendix Table A1. Interestingly, the community structures of site C in March and September deviate from their corresponding clusters and appear together in the rainy, hot season cluster. This observation is supported by the fact that water discharge of the Honghua dam results in similar bacterioplankton niches and a higher input of allochthonous organic matter during the rainy, hot season, providing nutrients for bacterioplankton (Brandão, Staehr, & Bezerra‐Neto, 2016). In addition, unweighted UniFrac and Bray–Curtis analyses revealed an enhanced dissimilarity between communities, suggesting that stratification determines the phylogenetic diversity in each community layer, as previously reported by Ávila et al. (2017) in two tropical shallow lakes in the Brazilian Atlantic Forest. Overall, our results suggest that spatial‐temporal variations in bacterioplankton community structure are shaped by hydro‐physicochemical variability relating to water temperature differences.

### 3.4 Relationship of bacterioplankton communities with hydro‐physicochemical properties

To explore the key drivers shaping bacterioplankton communities in the dammed Liu River, we provided comprehensive results using a variety of statistical methods. The partial Mantel test (permutations = 999) shows the significant effects of temperature on the bacterioplankton community (*p *<* *0.01) when pH and nutrition factors were controlled (Table 2). pH is significantly correlated with bacterioplankton communities (*r *=* *0.161, *p *=* *0.041) when the nutrition factor is controlled. DO is also significantly correlated with bacterioplankton communities (*p *<* *0.01) when temperature, pH, and nutrition factors are controlled.

**Table 2:** The influences of hydro‐physicochemical factors on bacterioplankton communities by a partial Mantel test

Effect of controlling for bacterioplankton community	Temperature				pH		Dissolved oxygen					
	pH		Nutrition		Nutrition		Temperature		pH		Nutrition	
	*r*	*p*	*r*	*p*	*r*	*p*	*r*	*p*	*r*	*p*	*r*	*p*
	0.263	0.001	0.307	0.001	0.161	0.041	0.331	0.001	0.395	0.001	0.407	0.001

**Figure 6:**
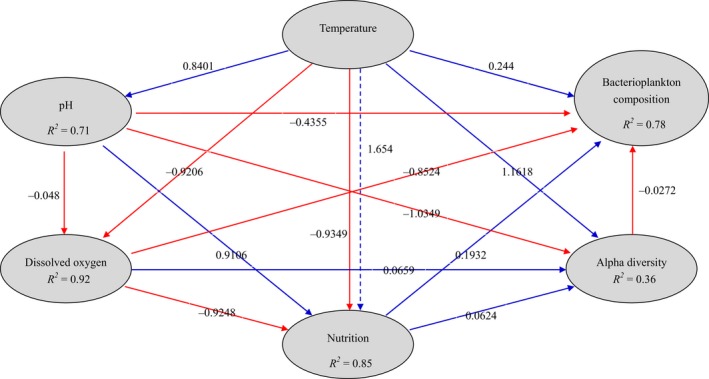
Directed graph of the PLS‐PM of temperature, pH, DO, and nutritional effects on bacterioplankton communities. Note: The path coefficients and the explained variability (*R* ^*2*^) were calculated after 999 bootstraps. The width of the arrows indicates the strength of the causal influence. Blue solid arrows indicate positive direct effects, red solid arrows indicate negative direct effects, and blue dashed arrows indicate positive indirect effects. Models with different structures were assessed using the GoF statistic, a measure of the overall prediction performance. For the model represented here, the GoF was 0.501

In addition, the PLS‐PM is represented here with a goodness‐of‐fit (GoF) value of 0.501 to integrate the complex interrelationships among environmental factors and bacterioplankton communities (Figure 6). According to the PLS‐PM, temperature, and nutrition exert direct positive effects on bacterioplankton composition and alpha diversity, and pH exerts direct negative effects on bacterioplankton composition and alpha diversity; however, DO exerts a direct negative effect on bacterioplankton composition and a direct positive effect on alpha diversity. Notably, temperature exerts significant positive or negative effects on pH, DO, and nutrition, which in turn cast the influences on bacterioplankton composition and alpha diversity (Figure 6). pH is a major environmental determinant shaping the patterns of bacterioplankton biodiversity and bacterioplankton community structures (Yun et al., 2016); however, we have very limited information about the patterns and processes by which overall bacterioplankton communities assemble across wide pH gradients in karst waters (Ren et al., 2015). DO exerts a direct negative effect on bacterioplankton composition, thus contributing to the shape of community structures of anoxygenic and oxygenic phototrophic bacteria in the dammed Liu River (Taipale, Jones, & Tiirola, 2009). It should be noted that pH, DO, and nutrition are affected by water temperature; consequently temperature plays pivotal roles in maintaining aquatic bacterial biodiversity patterns and bacterioplankton community composition, as previously reported by Wang, Pan, Soininen, Heino, and Shen (2016).

## 4 CONCLUSION

In the dammed Liu River, thermal regimes have been altered, which has resulted in considerable spatial‐temporal differences in TDS, Eh, DO, and pH that were strongly related to the Ca^2+^–Mg^2+^–HCO_3_^−^–SO_4_^2−^ water type and formed a different microenvironment for bacterioplankton. In this respect, the dominant bacterioplankton phyla Proteobacteria, Actinobacteria, Bacteroidetes, and Cyanobacteria account for 38.99%–87.24%, 3.75%–36.55%, 4.77%–38.90%, and 0%–14.44% of the total reads (mean relative frequency), respectively. The dammed Liu River is also populated by typical freshwater groups, such as *Brevundimonas, Novosphingobium, Zymomonas,* the Actinobacteria hgcI clade, the CL500‐29 marine group, *Sediminibacterium*,* Flavobacterium*,* Pseudarcicella*,* Cloacibacterium,* and *Prochlorococcus*, which covary with spatial‐temporal variations of hydro‐physicochemical factors. In addition, these groups played a key role in the carbon/nitrogen cycle and contributed to karst river metabolism. Temperature without clear water column thermal stratification plays pivotal roles in maintaining the hydro‐physicochemical factors and the aquatic bacterial biodiversity patterns in the dammed Liu River. This result highlights the concept that ecological niches for aquatic bacteria in dammed karst rivers do not occur accidentally but are the result of a suite of environmental forces.

## CONFLICT OF INTERESTS

None declared.

## AUTHOR CONTRIBUTIONS

SY, RXH, and QL conceived and designed the experiment. SY, RXH, AS, and QL performed the experiment. AS, ZJJ, YML, YDH, QL, XHW, WEGM, and JHC analyzed the data. YDH and QL led the writing of the manuscript. All authors contributed critically to the drafts and gave final approval for publication.

## ETHICS STATEMENT

None required.

## Data Availability

Raw sequence reads have been deposited to NCBI Sequence Read Archive under the accession number SRP126836.

## APPENDIX 1

### 1.1

**Table A1:** Mean alpha‐diversity of sampling site, specific depth and time

Sampling site	Chao1	Shannon	Simpson	Observed
A	376.93 ± 84.03 a	5.27 ± 0.45 b	0.93 ± 0.02 b	246.67 ± 48.22 a
B	376.53 ± 166.70 a	5.33 ± 0.70 b	0.93 ± 0.03 b	258.75 ± 108.09 a
C	419.04 ± 81.41 a	5.98 ± 0.28 a	0.96 ± 0.01 a	292.50 ± 58.58 a

Specific depth	Chao1	Shannon	Simpson	Observed
0	424.83 ± 133.68 a	5.61 ± 0.66 a	0.94 ± 0.03 a	282.11 ± 90.44 a
5 m	397.09 ± 111.23 a	5.56 ± 0.50 a	0.94 ± 0.02 a	270.38 ± 72.29 a
10 m	319.78 ± 65.90 a	5.16 ± 0.56 a	0.92 ± 0.03 a	223.83 ± 45.55 a

Time	Chao1	Shannon	Simpson	Observed
March	319.00 ± 62.86 b	5.12 ± 0.46 b	0.93 ± 0.02 a	206.78 ± 36.77 b
June	495.77 ± 125.02 a	5.97 ± 0.49 a	0.95 ± 0.02 a	337.63 ± 73.94 a
September	346.94 ± 32.17 b	5.36 ± 0.46 b	0.93 ± 0.03 a	247.17 ± 18.57 b

Values are the mean of analytical replicates for each sample ± standard deviations. Statistical pairwise multiple comparisons of data homogeneity were carried out by the Tukey test: means with the same letter in the same column are not significantly different at *P *<* *0.05.
